# Response to the Letter to the Editor Regarding “Serologic Survey of IgG Against SARS-CoV-2 Among Hospital Visitors Without a History of SARS-CoV-2 Infection in Tokyo, 2020–2021”

**DOI:** 10.2188/jea.JE20220202

**Published:** 2023-02-05

**Authors:** Takahiro Sanada, Michinori Kohara

**Affiliations:** Department of Microbiology and Cell Biology, Tokyo Metropolitan Institute of Medical Science, Tokyo, Japan

**Keywords:** SARS-CoV-2, COVID-19, IgG seroprevalence, hospital visitors, Tokyo

We are grateful to the Editor for this opportunity to respond to the comments in the letter to the Editor regarding our article, “Serologic Survey of IgG Against SARS-CoV-2 Among Hospital Visitors Without a History of SARS-CoV-2 Infection in Tokyo, 2020–2021”.^1^

The first issue addressed in the letter is the specificities of the kits used in our study. It is difficult to determine the actual sensitivity and specificity of the serological assay kits. For the purposes of our study, the specificities of two kits were presumed to be 100%, though the actual specificities might be lower. Additionally, the sensitivities of the combined results were not determined. Therefore, we cannot exclude the possibility of contamination by false-positive and false-negative results. As Lyu et al mentioned, our results must be interpreted with care. Optimization of cutoff values and/or the combined use with other antibody detection kits is expected to provide greater reliability for the results.

The second issue addressed in the letter is the single positive samples. To detect anti-SARS-CoV-2 antibodies, we used two kits and combined the results. Although the antibody kinetics in symptomatic patients with coronavirus disease 2019 (COVID-19) have been reported in several studies,^2^^,^^3^ the antibody kinetics against each SARS-CoV-2 protein in asymptomatic infections remain poorly understood. Lei et al reported that the antibody levels in asymptomatic infections were lower than those in symptomatic infections, with the immunoglobulin G (IgG) response declining or becoming undetectable in 2 months.^4^ Single positive cases may reflect low antibody levels. Further analysis of the antibody kinetics in asymptomatic infections will be needed. Lyu et al also mentioned possible contamination resulting from vaccinated participants. Notably, our study assessed the seroprevalence of anti–SARS-CoV-2 IgG among 23,234 visitors to Tokyo-area hospitals during the interval from September 2020 through March 2021. According to the Tokyo Metropolitan Government, as of April 12, 2021, only 418 residents of Tokyo had been vaccinated.^5^ We, therefore, believe vaccination would have little influence on seroprevalence in our study.

The third issue addressed in the letter is the method used to detect anti-N protein antibody. We used an iFlash 3000 chemiluminescence immunoassay analyzer (Shenzhen YHLO Biotech, Shenzhen, China) with an iFlash-SARS-CoV-2 IgG-N kit (YHLO Biotech).

We thank the authors of this letter for their comments and suggestions.
